# On the Protective Influence of Vaccination

**Published:** 1844-11-16

**Authors:** 


ON THE PROTECTIVE INFLUENCE OF VACCINATION.

The general conclusion, drawn by Dr. Retzius, of Stockholm, from his observations of small-pox, and the effects of vaccination in Sweeden, are these: —'The protection afforded by vaccination from the close of the second year of life against the contagion of the variolous poison, usually lasts unimpaired to the end of the thirteenth year or so; after this period it begins to lose its effect, and gradually becomes more and more uncertain on to the twentieth or twenty-first year of life. For the next four or five years, the disposition to the small-pox seems almost to have recovered its original integrity; and this state of liablity continues unimpaired up to the age of forty years or so. At about this epoch of life it begins to approach nearer and nearer to the limit of its existence —which it reaches, in the majority of cases, about the fiftieth year—the period when the general revolution of the human body commences to take place.'

The practical inference to be drawn from these remarks is the propriety of repeating vaccination in about thirteen or fourteen years after the first performance. This advice is in accordance with the observations of the most experienced practitioners: it would be well if it were more generally acted upon.—*Medico-Chirurgical Review.*

